# IL-17A Monoclonal Antibody Partly Reverses the Glucocorticoids Insensitivity in Mice Exposed to Ozonec

**DOI:** 10.1007/s10753-017-0523-7

**Published:** 2017-02-13

**Authors:** Xia Fei, Peng-yu Zhang, Xue Zhang, Guo-qing Zhang, Wu-ping Bao, Ying-ying Zhang, Min Zhang, Xin Zhou

**Affiliations:** Department of Respiratory Medicine, Shanghai General Hospital, Shanghai Jiao Tong University, No. 100, Haining Road, Shanghai, 200080 China

**Keywords:** interleukin-17A, ozone, airway inflammation, glucocorticoids insensitivity

## Abstract

Exposure to ozone has been associated with airway inflammation and glucocorticoid insensitivity. This study aimed to observe the capacity of anti-murine interleukin-17A monoclonal antibody (IL-17mAb) to reverse ozone-induced glucocorticoid insensitivity and to detect its effects with glucocorticoids in protecting against airway inflammation. After C57/BL6 mice were exposed to ozone (2.5 ppm; 3 h) for 12 times over 6 weeks, PBS, IL-17mAb (50 ug/ml), dexamethasone (2 mg/kg), and combination administration of IL-17mAb (50 ug/ml) and dexamethasone (2 mg/kg) were intraperitoneally injected into mice at a dose of 0.1 ml, respectively, for 10 times over 5 weeks. At sacrifice, lung histology, airway inflammatory cells, levels of related cytokines in bronchoalveolar lavage fluid (BALF), and serum were analyzed, airway inflammatory cell infiltration density and mean linear intercept (Lm) were measured, the expression of IL-17A mRNA, glucocorticoid receptors (GR), NF-κB, and p38 mitogen-activated protein kinase (MAPK) phosphorylation were determined. We found that combination administration markedly reduced ozone-induced total inflammatory cells, especially neutrophils; inhibited levels of cytokines, including IL-8, IL-17A, and TNF-α in BALF; and suppressed airway inflammatory cell infiltration density and Lm. Additionally, combination administration significantly elevated levels of IFN-γ in BALF, decreased the dexamethasone-induced increase of IL-17A mRNA, and increased the expression of GR and decrement of NF-κB and p38MAPK phosphorylation, which are also related to glucocorticoids insensitivity. Collectively, combination administration shows profound efficacy in inhibiting certain cytokines, and IL-17 mAb partly improved the glucocorticoids insensitivity *via* modulating the enhanced production rate and improving expression of IL-17A induced by glucocorticoids administration and p38MAPK, NF-κB signaling pathway.

## BACKGROUND

Ozone (O_3_) is an exogenous oxidant which adversely affects human health by irritating the mucosa and harming the respiratory system [2]. Oxidative stress and its products are involved in the mechanism underlying ozone-induced inflammation, induce and amplify the bronchial hyperresponsiveness [3, 14]. Oxidative stress is also a feature of the airways, resulting from the release of reactive oxygen and nitrogen species from inflammatory and immune cells in the airways [14], and plays an important role in the pathogenesis of chronic obstructive pulmonary disease (COPD) [13, 40, 42] and in the induction of glucocorticoids insensitivity. The mechanisms and pathways by which oxidative stress can lead to chronic inflammation and emphysema have been investigated in mouse models of cigarette exposure [44, 48]. Furthermore, direct exposure of mice to an oxidant gas, ozone, results in emphysema and chronic lung inflammation reminiscent of COPD [50]. Experimental ozone exposure at high concentrations can also induce bronchial hyperresponsiveness resulting from an increase in contractility of the airways [27, 45].

Glucocorticoids, characteristic of anti-inflammatory and immunosuppressive actions [10, 41], are the mainstay for the treatment of chronic inflammatory diseases including asthma and COPD. However, it has been recognized that certain patients do not respond well to glucocorticoids treatment and at high risk of adverse effects [5, 22]. Several mechanisms may underlie the reduced glucocorticoids sensitivity, namely, glucocorticoids insensitivity, which is influenced by multiple factors [8], including the role of the mitogen-activated protein kinases (MAPK) [12, 28], defective histone acetylation, and GR modification [6]. Glucocorticoids insensitivity is also a feature of other immune and inflammatory disease, including rheumatoid arthritis, inflammatory bowel disease, and systemic lupus erythematosis [6].

Interleukin (IL)-17, also known as IL-17A, is produced by CD4^+^ Th17 cell [46], cytotoxic T cells, invariant natural killer T cells [33], lymphoid tissue-inducer like cells [47, 53], and CD8^+^ T cells [11]. IL-17A induces the release of the pro-inflammatory cytokines, IL-8, CXCL1, KC, GCSF, and GM-CSF from the airway epithelial cells, smooth muscle cells, and macrophages and thereby orchestrates neutrophilic inflammation and release of reactive oxygen species [53, 56]. The previous investigations also showed that IL-17A contributed to steroid insensitivity in patients with severe asthma [54]. Thus, IL-17A may be involved in corticosteroid responses to oxidant stress and IL-17A expression may underlie glucocorticoids insensitivity found in patients with severe asthma and COPD. However, few investigations have been conducted to determine whether the oxidant-mediated disruption of the combination administration of IL-17mAb and glucocorticoids contributes to reversing the corticosteroid insensitivity.

In regard to the high risk of adverse effects of glucocorticoids including diabetogenesis, osteoporosis, muscle wasting, skin thinning and weight gain [1, 30], and the above noted corticosteroid insensitivity, more effective anti-inflammatory therapeutic approaches are needed to explore. So, we used a mouse model of chronic exposure to ozone that leads to airway inflammation and lung destruction, to investigate whether IL-17mAb can overcome the glucocorticoids insensitivity.

## METHODS

### Mice and Ozone Exposure

Pathogen-free, 10∼12-week-old male C57/BL6 mice, obtained from Shanghai Laboratory Animal Center, were housed within filter-topped cages, maintained in a controlled temperature (19∼23 °C) and humidity (40∼60%), facility with a strict 12-h light-dark cycle and were given free access to food and water. According to random number table, mice are divided into four parts: ozone–exposed + PBS-treated model, ozone–exposed + PBS-treated + dexamethasone-treated model, ozone-exposed + IL-17 mAb-treated model, ozone–exposed + IL-17mAb-treated + dexamethasone-treated model. Animals were exposed to ozone produced from an ozoniser (Model 500 Sander Ozoniser, Germany), mixed with air, for 3 h at a concentration 2.5 parts per million (ppm) in a sealed Perspex container, twice a week for 6 weeks. Control animals received medical air only over the equivalent period. Ozone concentration was continuously monitored with an ozone probe (ATi Technologies, Ashton-U-Lyne, UK). From day 42, mice were injected intraperitoneally with IL-17mAb (2 mg/kg, 0.1 ml), dexamethasone (2 mg/kg, 0.1 ml), or vehicle 1 h before ozone exposure for 10 times. All the animal experiments were strictly conducted in accordance with the protocols approved by the Ethics Committee for Animal Studies at Shanghai General Hospital, China. All surgery was performed under sodium pentobarbital anesthesia, and all efforts were made to minimize suffering.

### Bronchoalveolar Lavage Fluid (BALF) and Cell Counting

Immediately after the assessment of airway reactivity, mice were sacrificed after anesthesia with an overdose of pentobarbitone (500 mg/kg intraperitoneally). The tracheal was exposed and intubated with PE-60 tubing (0.72-mm-inner diameter, 1.22-mm-outer diameter). BALF samples were obtained as described previously [50]. Briefly, mice were lavaged with three aliquots of 0.3 ml sterilized saline and BALF was retrieved. Return volume was recorded and was consistently >80% of the instilled volume. The BALF was then centrifuged at ×1500*g* for 10 min at 4 °C. The supernatant was stored at −80 °C for further assay. Total differential cell counts were determined under a microscope. The remaining cell pellet was resuspended in 1 ml PBS solution. Total cell counts were determined using a haemocytometry, by adding 100 μl of the cell suspension to 100 μl trypan blue stain. Differential cell counts were performed on cytocentrifuge preparations (Cytospin 2; Shandon, UK) stained with Wright-Giemsa by counting approximately 400 cells under ×400 magnification from each individual of four different random locations by two independent, blinded investigators.

### Bronchoalveolar Lavage and Measurements of BALF Cytokines

All the cytokines, including interleukin (IL)-8, IL-17A, interferon (IFN)-γ in both supernatants of BALF and serum, and tumor necrosis factor (TNF)-α in BALF were determined by enzyme-linked immunosorbent assay (ELISA), as previously described [58]. Measurements of IL-8, IL-17A, IFN-γ, and TNF-α concentrations were performed in lung homogenate supernatants with commercial available ELISA kits (R&D Systems China Co., Ltd., Shanghai, China) and were performed according to manufacturer’s instructions.

### Histological and Morphometric Analysis

After BALF, the left lung lobe was removed and fixed in 10% neutral-buffered formalin solution and later embedded into paraffin. The lungs were then dissected and placed in fresh paraformaldehyde for 48 h. Routine histological techniques were used to paraffin-embed the tissue, 4-**μm** paraffin sections were placed onto Fisher PLUS slides. After deparaffinization and rehydration, 5 μm sections of the lung tissue were stained with hematoxylin–eosin (HE), dehydrated, and mounted.

The mean linear intercept, a measure of interalveolar septal wall distance, was determined using a reticule with a Thurlbeck grid comprising of 5 lines (each 550 mm long), with 10 fields per section assessed at random. Two slides per mouse were coded and analyzed using a reproducible scoring system described elsewhere [26]. Fields with airways or vessels were avoided by moving one field in any one direction. Linear intercept (Lm) was calculated by dividing the length of the line by the number of alveolar wall and grid line interceptions. All counts were studied by two independent observers in a blinded fashion.

Digital image analysis was performed on histological sections, using Image-Pro Plus software version 5.0 (Media/cybernetics, Silver Springs, MD, USA), nuclear profiles in HE-stained sections were counted in the lamina propria. The severity of inflammatory response was expressed as the ratio of area of the cells to the whole brochial surface area.

### Real-time Reverse Transcription-Polymerase Chain Reaction

RNA extracted from frozen stored the lung tissue which collected at the time of dissection using an RNeasy Mini kit (Qiagen). RNA yield was then amplified *via* PCR using an Omniscript Reverse Transcriptase kit (Qiagen) and stored at −80 °C until required. 0.5 μg per sample of RNA was used to synthesize single-stranded complimentary DNA (cDNA) using High Capacity cDNA Reverse Transcription Kit (Applied Biosystems, CA, USA) in a PTC-200 Peltier Thermal Cycler (MJ Research, Watertown, Mass., USA) according to manufacturer instructions. The cDNA was synthesized using the energic Scriptc DNA synthesis kit (ShineGene Co., Ltd., Shanghai, China). Real-time quantitative PCR (RT-qPCR) was performed with a SYBR Kit (Bioline). IL-17A mRNA was quantitated by real-time PCR (7300 Real-Time PCR Systems; Applied Biosystems, Carlsbad, CA) using intron-spanning primers (IL-17A sense, 5′-CCAGGGAGAGCTTCATCTGT-3′, and antisense, 5′-AGGAAGTCCTTGGCCTCAGT-3′) and SYBR-green detection. Cycling conditions were as follows: step 1, 15 min at 95 °C; step 2, 20 s at 94 °C; step 3, 20 s at 55 °C; and step 4, 20 s at 72 °C, repeating step 2 to step 4, 55 times. RT-PCR results were analyzed with the ΔΔCT method [36]. Gene expression was expressed as a ratio of the gene of interest mRNA to GAPDH mRNA.

### Western Blot Analysis

The lung tissues were homogenized using 1.4 mm Precellys Ceramic beads and Precellys 24 homogenizer (Peqlab, Erlangen, Germany) at 6800 rpm for 15 s and cytosolic proteins were extracted with a hypotonic buffer (active motif, part #100505) and detergent (active motif, part #100512) by centrifugation at 14000 rpm for 30 s at 4 °C and qualified by bicinchoninic acid assay analysis. Equal amounts of protein were separated by SDS-PAGE and electrophoretically transferred to nitrocellulose membrane and then incubated with primary antibodies against phospho-p38 MAPK, total p38 MAPK, GR, and NF-κB (Cell Signaling Technology, Beverly, CA) for blot detection. Final protein concentration was determined using a protein assay.

### Statistical Analysis

Data are expressed as mean ± SEM. The statistical analysis and graphics were performed using GraphPad PRISM, version 5.0 (GraphPad Software, San Diego, CA). One-way ANOVA with Bonferroni’s post hoc test (for equal variance) or Dunnett’s T3 post hoc test (for unequal variance) was performed for comparisons among multiple groups. *P* < 0.05 was considered significant.

## RESULTS

### Total and Differential Cell Counts of BALF

**Fig. 1:**
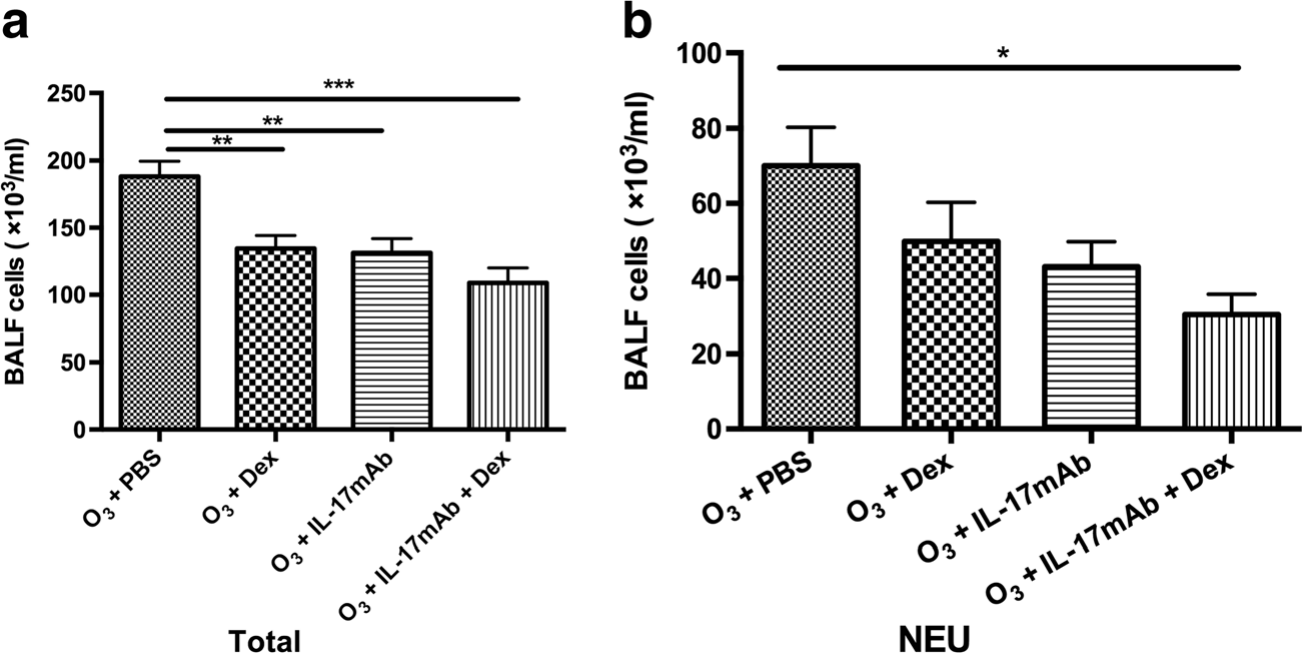
Mean numbers of total cells and neutrophils recovered from bronchoalveolar lavage fluid (BALF) of ozone-exposed mice. *P < 0.05, **P < 0.01, and ***P < 0.001, *NEU* neutrophils.

BALF was collected 24 h after the last airway challenge of ozone-induced mice. As expected, compared with PBS-treated ozone-exposed controls, numbers of total cells in BALF were decreased significantly at 48 h after Dex treatment, IL-17mAb treatment or vehicle compared with the numbers after PBS-treated (*P* < 0.01, *P* < 0.01, and *P* < 0.001, respectively) (Fig. 1a). Animals treated with Dex and IL-17mAb showed a significant decrease compared with Dex or IL-17mAb treatment alone in total cell counts in BALF, but there was no statistical significance (Fig. 1a). However, for neutrophils, only combined administration of IL-17mAb and dexamethasone on ozone-exposed mice demonstrated significantly decreased in counts in BALF compared with PBS-treated ozone-exposed mice (*P* < 0.05) (Fig. 1b).

### BALF and Serum Cytokine Levels

**Fig. 2:**
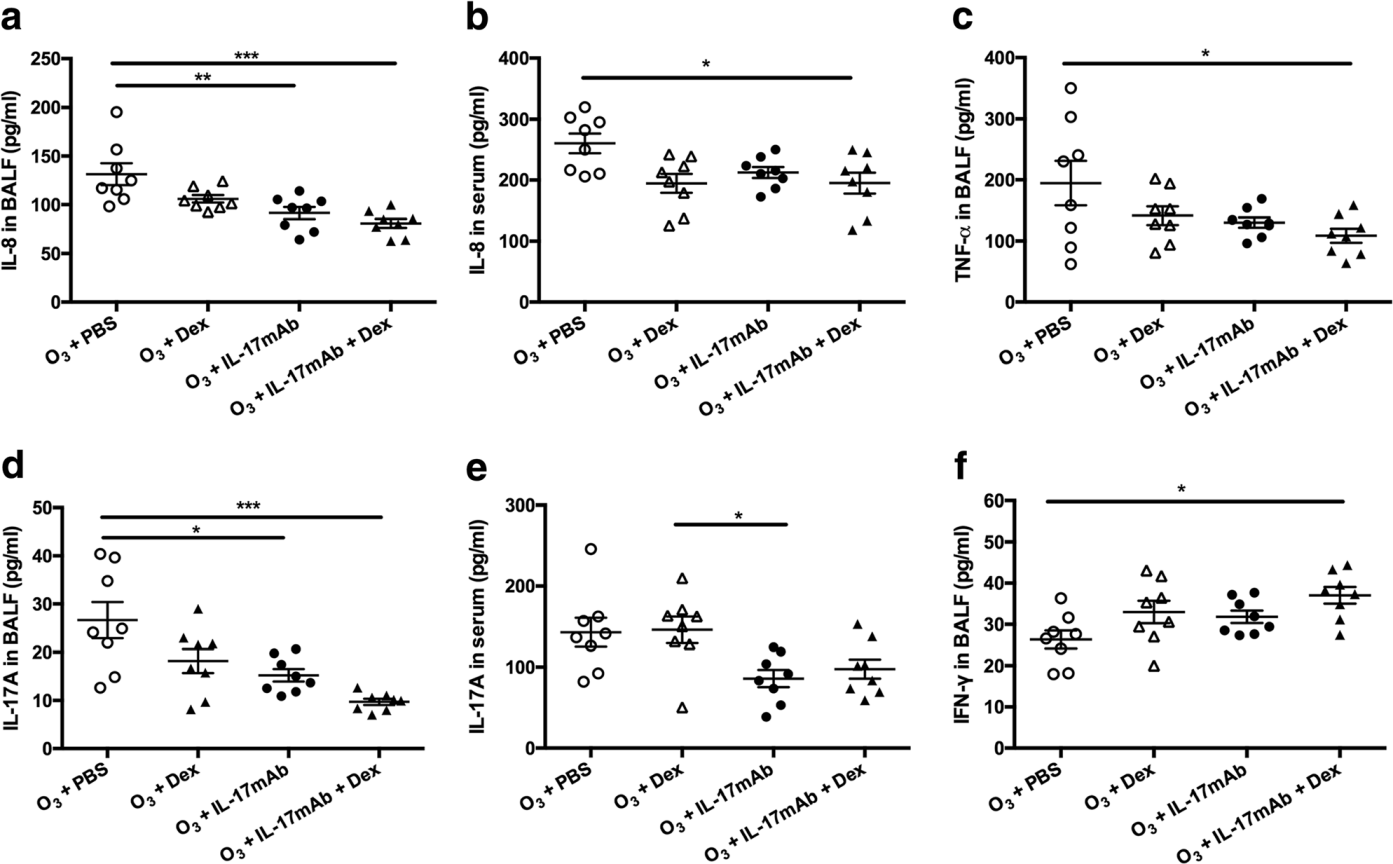
Mean levels of IL-8 (**a** in BALF and **b** in serum), TNF-α in BALF (**c**), IL-17A (**d** in BALF and **e** in serum), and IFN-γ in BALF (**f**) of ozone-exposed mice. *P < 0.05, **P < 0.01, and ***P < 0.001.

**Fig. 3:**
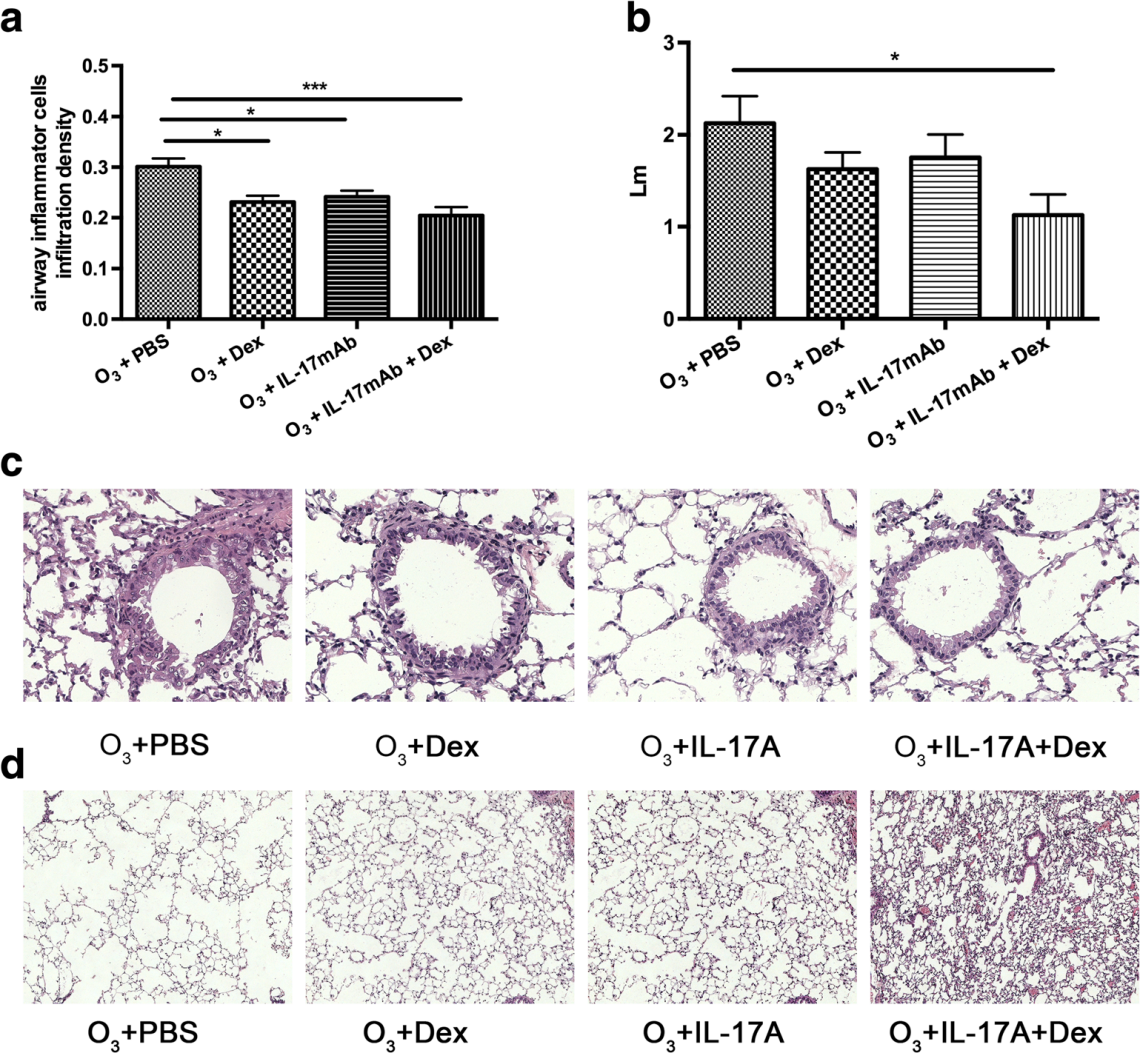
Mean value of airway inflammatory cell infiltration density in the lungs of ozone-exposed mice (**a**). Mean linear intercept (Lm) in the lungs of ozone-exposed mice (**b**). *P < 0.05, **P < 0.01, and ***P < 0.001. Representative histological sections of airways with inflammatory cell infiltration (**c**) (×400) and enlargement of alveolar spaces (**d**) (×100).

Ozone exposure in vehicle-treated mice evoked significant decreases in IL-8 (in BALF and serum) (*P* < 0.001 and *P* < 0.05) (Fig. 2a, b). Similarly, IL-17mAb-treated mice exposed to ozone exhibited significant decrease in IL-8 in BALF compared with PBS-treated ozone-exposed controls (*P* < 0.01) (Fig. 2a). There were significant differences in IL-17A in BALF after Dex treatment, IL-17mAb treatment, or vehicle compared with mice exposed to ozone after PBS-treated. Administration of IL-17mAb and Dex or vehicle suggested significant reduction in IL-17A in BALF compared with PBS-treated mice (*P* < 0.001) (Fig. 2d). Whereas Dex-treated ozone-exposed mice showed levels of IL-17A in serum were no significantly changed compared with PBS-treated ozone-exposed mice. Moreover, combined administration of IL-17mAb and dexamethasone showed the inhibition of the levels of IL-17A induced by IL-17mAb (Fig. 3e). As we hypothesized previously, compared with the PBS-treated controls, mice in the combined administration of IL-17mAb and dexamethasone model exhibited the lower levels of TNF-α (*P* < 0.05) (Fig. 2c). Whereas IL-17mAb and Dex-treatment vehicle model showed higher levels of IFN-γ in BALF than PBS-treated animals (*P* < 0.05) (Fig. 2f), but there was no significant difference in treatment effect between Dex-treated and vehicle models (Fig. 2f).

### The Airway Inflammatory Cell Infiltration Density and the Emphysema Score

There were significant differences in airway inflammatory cell infiltration density and the emphysema score in the vehicle-treated models and the PBS-treated ozone-exposed controls. In the ozone-exposed vehicle-treated mice, airway inflammatory cell infiltration density and the emphysema score were significantly lower than the ozone-exposed PBS-treated mice (*P* < 0.001, *P* < 0.05, respectively) (Fig. 3a, b). In addition, the airway inflammatory cell infiltration density significantly decreased ozone-exposed mice with IL-17mAb treatment only compared with PBS-treated mice (*P* < 0.05, *P* < 0.05) (Fig. 3a). In comparison with IL-17mAb or Dex only, the emphysema score was not significantly different from PBS-treated mice (Fig. 3b).

### IL-17A mRNA Levels in Lung Tissue

**Fig. 4:**
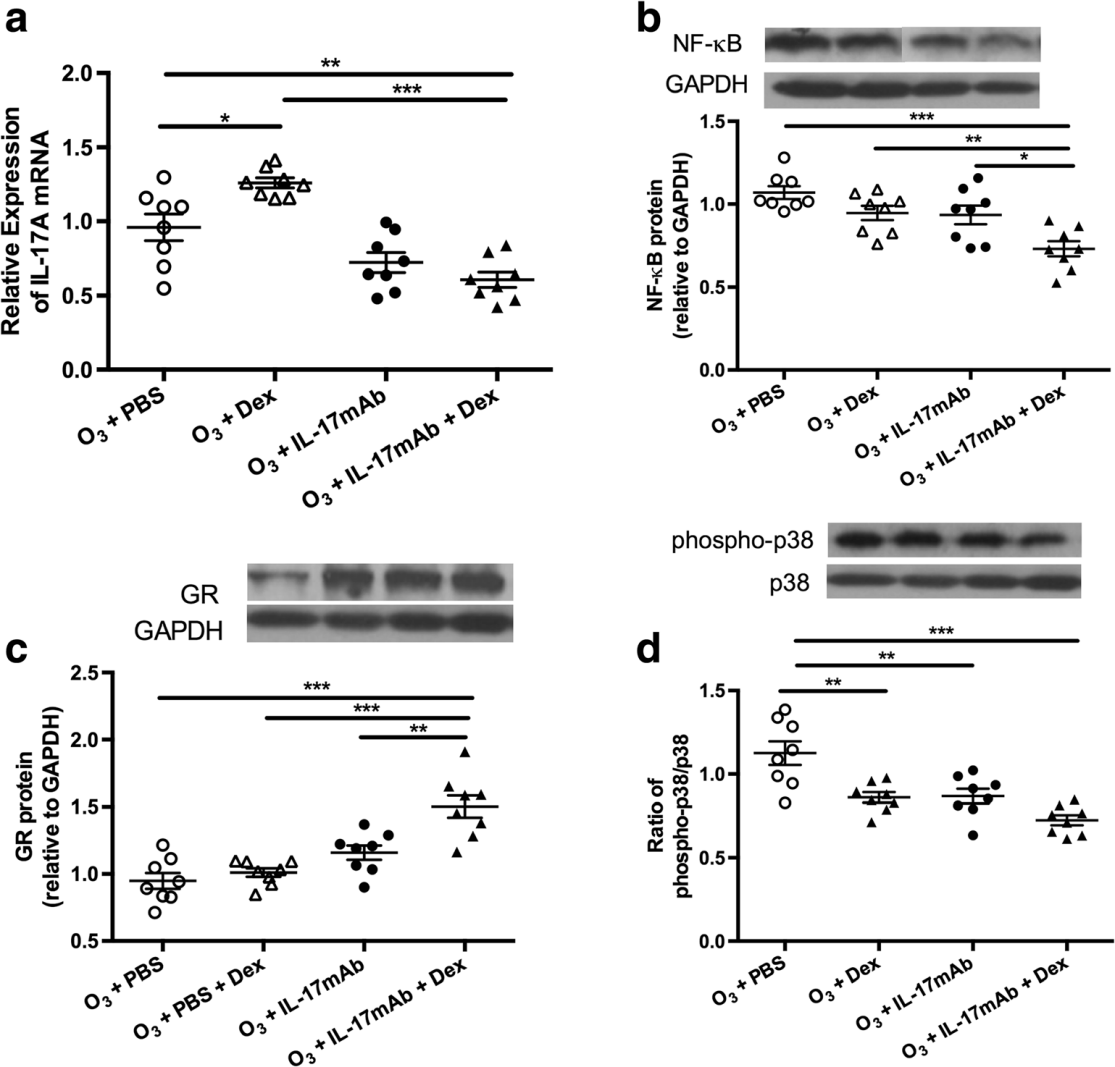
Expression of IL-17A mRNA (**a**) in the lung tissue of ozone-exposed mice. Western blot analysis of ratios of NF-κB (**b**), glucocorticoid receptors (GR) (**c**), and phosphorylated p38 MAPK to total P38 MAPK (**d**) in the lung tissue in four groups of ozone-exposed mice. *P < 0.05, **P < 0.01, and ***P < 0.001.

In the mice exposed to ozone, the mRNA levels of IL-17A in the vehicle-treated mice were significantly decreased compared with PBS-treated mice (*P* < 0.01) (Fig. 4a). However, In the Dex-treated mice exposed to ozone, the expression of IL-17A mRNA was higher compared with PBS-treated mice (*P* < 0.05) (Fig. 4a). After the vehicle-treatment, mice exposed to ozone exhibited significant decrease in the mRNA levels of IL-17A in comparison with Dex-treated mice exposed to ozone (*P* < 0.001) (Fig. 4a).

### The Gene and Protein Expression of NF-κB and GR and p38 MAPK Phosphorylation

Combined administration of IL-17mAb and dexamethasone on ozone-exposed mice significantly decreased the expression of NF-κB compared with PBS-treated mice, Dex-treated mice, IL-17mAb mice (*P* < 0.001, *P* < 0.01, and *P* < 0.05, respectively) (Fig. 4b), but not between PBS-treated or Dex-treated or IL-17mAb mice exposed to ozone and PBS-treated mice exposed to ozone (Fig. 4b). Similarly, compared with PBS-treated mice, Dex-treated mice, and IL-17mAb mice, combined administration of IL-17 mAb and dexamethasone on ozone-exposed mice significantly increased the expression of GR (*P* < 0.001, *P* < 0.001, *P* < 0.01, respectively) (Fig. 4c).

The p38MAPK phosphorylation was significantly decreased in combined administration of IL-17mAb and dexamethasone models as well as Dex-treated mice and IL-17mAb mice (*P* < 0.001, *P* < 0.01, and *P* < 0.01, respectively) (Fig. 4d). However, combined administration of IL-17mAb and dexamethasone on ozone-exposed mice had a lower level of p38MAPK phosphorylation than Dex-treated mice and IL-17mAb mice but was not significantly changed (Fig. 4d).

## DISCUSSION

The novel points in our current work are as follows: on the one hand, though the inhibition of monotherapy of IL-17mAb in ozone-induced airway inflammation has been investigated, combination administration of IL-17mAb and dexamethasone was used for the first time to demonstrate its combined effects on inhibiting ozone-induced airway inflammation and provided profound suppression of a range of inflammatory mediators produced by ozone exposure; on the other hand, we surprisingly found that glucocorticoid insensitivity may due to the potential increase in the production and transcription of IL-17A induced by glucocorticoids.

Glucocorticoids suppress inflammatory gene transcription by forming a complex with the glucocorticoid receptor (GR) that inhibits the function of transcription factors such as nuclear factor (NF)-κB, a process known as transrepression [17]. Down-regulation of the expression of GR results in glucocorticoids insensitivity and up-regulation of the expression of GR could partly reverse glucocorticoids insensitivity. Glucocorticoids insensitivity may result from the reduced numbers and the attenuated activation of GR. Some cytokines such as TNF-α and IL-1 are known to down-regulate GR expression and attenuate the cell’s response to steroids [35, 52]. The involvement of p38MAPK is associated with ozone exposure on the response to glucocorticoid in a mouse model of asthma and inhibiting the phosphorylation of p38MAPK could improve the response to glucocorticoid and reverse the airway inflammation [4]. IFN-γ reverses steroid response *via* inhibition of p38 MAPK pathway. Inhibiting p38MAPK may potentially reverse steroid insensitivity. IFN-γ could be a potential inhibitor of cytokine-induced p38MAPK activation and that IFN-γ is critical to maintain corticosteroid sensitivity [18]. Our study presented that combination administration significantly enhanced the expression of GR and IFN-γ, decreased the expression of TNF-α, p38MAPK, and NF-κB. But monotherapy did not alter the expression of IFN-γ and TNF-α.

There are various indications that IL-17A may be involved in the glucocorticoids insensitivity [37, 59]. IL-8 [7, 34] and IL-17A [43] contribute to the recruitment of neutrophils. To our knowledge, IL-17A can be found in the human sputum, BALF, and peripheral blood. Increasing evidence suggests that IL-17A significantly stimulates neutrophil maturation, migration, and function, and acts directly on the epithelial cells, airway fibroblasts, and smooth muscle cells to induce the production of chemokines and other cytokines, such as TNF-α, which recruit neutrophils and monocytes into the airways and lung which promote and worsen the neutrophilic inflammation status [9, 19, 21, 24, 29, 39]. Lung neutrophils show reduced expression of the glucocorticoid receptors [38]. The effects of glucocorticoids on cytokine production from airway neutrophils are reduced. Increased numbers of airway neutrophils lacking GR may contribute to glucocorticoid resistance in COPD patients. Inflammation itself might contribute to reduced glucocorticoids sensitivity [51]. Moreover, Th17-induced neutrophilic airway inflammation in mice was reported to be glucocorticoids insensitive [32]. IL-17A reduced HDAC activity, overexpression of HDAC2 reversed IL-17A-induced glucocorticoids insensitivity [59], in other words, inhibiting the expression of IL-17A, in turn increasing HDAC2 activity, and glucocorticoids insensitivity will be partly halted. The reduction of HDAC2 activity contributes to the transcription of NF-κB and enhances the activities of proinflammatory cytokines such as IL-8 and TNF-α [20]. TNF-α is a potent pro-inflammatory cytokine released by the cells of the immune system upon stimulation [49] and is associated with GR and points to an intricate interplay with GR signaling [51]. There are previous data from COPD alveolar macrophages that IL-8 is glucocorticoids insensitivity [15, 16].

There are previous data from COPD alveolar macrophages that IL-17A induced IL-8 production is glucocorticoids insensitivity [15, 16, 21]. These previous studies add further weight to another finding that macrophage IL-8 production is glucocorticoids insensitive [23]. Prior studies have showed that IL-17A increases the release of IL-8 from the bronchial epithelial, and IL-17A may increase human neutrophil recruitment through IL-8 *in vitro*. IL-17A can also stimulate the production of pro-inflammatory cytokines IL-1β and TNF-α, which can synergize with IL-17A [25, 31, 43]. These modifications and various processes regulated by IL-17A are shown to be involved in the glucocorticoids insensitivity, but it is still unknown whether there might be a direct relationship exists between IL-17A and glucocorticoids insensitivity.

In our study, we determined whether the administration of IL-17mAb affects the response of corticosteroids on chronic lung inflammation and emphysema induced by ozone exposure. Remarkably, we conclude that dexamethasone had no effect in altering the number of neutrophils, Lm, IL-8, IL-17A, TNF-α, IFN-γ, GR, and the increased expression of NF-κB induced by chronic ozone exposure. Some effects of dexamethasone were observed on the number of total inflammatory cells and the airway inflammatory cell infiltration density, but it is also observed in IL-17mAb treated group. In addition, we have also shown that glucocorticoids induced the increase of the production of IL-17A and the expression of IL-17A mRNA induced by chronic exposure to ozone. Combined administration of IL-17mAb and dexamethasone on ozone-exposed mice significantly decreased the expression of IL-17A mRNA and NF-κB compared with monotherapy of dexamethasone, which was consistent with reduced pro-inflammatory cytokine production and increased GR expression. These data may therefore strengthen the view that a potential increase in the production of systemic IL-17A and the expression of IL-17A gene transcription may be a cause of corticosteroids insensitivity and account for IL-17A inhibitor partly reversing the glucocorticoids insensitivity. This could also indirectly explain why glucocorticoids do not work well in certain COPD populations suggesting reduced sensitivity.

Compared with the monotherapy responses, the reduced inflammatory cytokines (IL-8, IL-17A, and TNF-α) have been observed after the combined administration of IL-17mAb and dexamethasone. Moreover, neutrophils in BALF were not significantly altered after dexamethasone or IL-17mAb treatment alone. However, combined administration of IL-17mAb and dexamethasone on ozone-exposed mice exhibited a significant decrease in the total inflammatory cells and neutrophils in BALF and significantly decreased the airway inflammatory cell infiltration density than the single IL-17mAb treated group compared with the PBS model. In addition, we observed that in the chronic exposure model to an oxidant, ozone, the combination administration of IL-17 mAb, and dexamethasone ameliorates the induction of emphysema, and this was not seen in the monotherapy of dexamethasone or IL-17mAb response, which is in agreement with our previous work [57]. This indicated that IL-17A was not involved in the induction of emphysema but contributed to increase the effect of corticosteroids on attenuating the emphysema.

To further investigate for a special effect of combined administration, we detected the Th1-driven cytokine IFN-γ in BALF. Here, we demonstrated that combination administration increased the levels of IFN-γ, a prominent product of CD8^+^ cells, but decreased the induction of emphysema with alveolar enlargement, which is likely to be inconsistent with the previous studies that overexpression of IFN-γ led to emphysema and enhanced neutrophil-rich inflammation in the adult murine lung [55]. Nevertheless, whether and how IFN-γ modulates emphysema and inflammation needs further investigation.

Combined with the previous work we have finished [57], we have shown that chronic exposure to ozone induces lung emphysema and inflammation as previously described, and in the present study, we also demonstrated the fact that in glucocorticoid-treated mice exposed to ozone, the level of systematic IL-17A was higher, compared with that of the controls, which was different from what was previously reported. Similarly, we also confirmed the finding in the expression of IL-17A mRNA.

As mentioned above, we concluded that IL-17 mAb was comparable to glucocorticoids in the role of certain anti-inflammation, and combination administration of IL-17mAb and glucocorticoids have profound anti-inflammatory effects on ozone-induced airway inflammation and partly restore glucocorticoid sensitivity (reestablish the beneficial effects of glucocorticoids). For IL-17A inhibitors in clinical development, these data provide a strong rational for combination trials with glucocorticoids and provide partial benefit in reversing the glucocorticoids insensitivity.

## Abbreviations

COPD: Chronic obstructive pulmonary disease
Dex: Dexamethasone
BALF: Bronchoalveolar lavage fluid
IFN: Interferon
IL: Interleukin
mAb: Monoclonal antibody
mRNA: Messenger ribonucleic acid
PBS: Phosphate-buffered saline
RT-PCR: Reverse transcription–polymerase chain reaction
TNF: Tumor necrosis factor
MAPK: Mitogen-activated protein kinase
NF-κB: Nuclear factor kappa B
GR: Glucocorticoid receptors.
